# Modeling Relief Demands in an Emergency Supply Chain System under Large-Scale Disasters Based on a Queuing Network

**DOI:** 10.1155/2014/195053

**Published:** 2014-02-06

**Authors:** Xinhua He, Wenfa Hu

**Affiliations:** ^1^School of Economics Management, Shanghai Maritime University, Shanghai 201306, China; ^2^School of Economics and Management, Tongji University, Shanghai 200092, China

## Abstract

This paper presents a multiple-rescue model for an emergency supply chain system under uncertainties in large-scale affected area of disasters. The proposed methodology takes into consideration that the rescue demands caused by a large-scale disaster are scattered in several locations; the servers are arranged in multiple echelons (resource depots, distribution centers, and rescue center sites) located in different places but are coordinated within one emergency supply chain system; depending on the types of rescue demands, one or more distinct servers dispatch emergency resources in different vehicle routes, and emergency rescue services queue in multiple rescue-demand locations. This emergency system is modeled as a minimal queuing response time model of location and allocation. A solution to this complex mathematical problem is developed based on genetic algorithm. Finally, a case study of an emergency supply chain system operating in Shanghai is discussed. The results demonstrate the robustness and applicability of the proposed model.

## 1. Introduction

In last decades a number of natural or manmade disasters such as earthquakes, volcanoes, floods, hurricanes, epidemics, explosions, fires, and violent attacks have risen threefold and death counts and property losses are reported in every disaster [1, 2]. Although most of those disasters were not avoided, efficient delivery of emergency supplies could save lives and reduce loss. Those ubiquitous disasters have aroused intensive concerns of emergent relief demanding. Emergency relief requires coordinated and rapid responses and supplies.

The emergency supply chain (ESC) is to locate points of emergency equipment and supplies and to relieve those in need with food, water, shelters, and medical care promptly [3]. In fact, ESC is a network of combined organizations mutually and cooperatively to plan, manage, and control the flow of emergency commodities for the purpose of maximizing the affected human survival rate and minimizing the cost of the rescue actions after disasters.

ESC is a typical three-echelon network: supply points, emergency logistics centers, and demand points. The key challenges to ESC as compared to the business supply chain are highlighted as: follows demand and route uncertainties, complex communication and coordination, timely delivery, and limited resources [4, 5]. Those uncertainty challenges in disaster characteristics were addressed in previous literatures through the use of probabilistic models [6], queuing theory [7], and fuzzy methods [8].

The objectives of the ESC system are to improve the performance while minimizing the response time. The response time includes transportation time, waiting time, and service time of relief commodities in case of congestions. Queuing models are effective measures to calculate the queue length, sojourn time, and waiting time and probabilities of any delay, idleness, and turnaways due to insufficient waiting accommodation [9]. Therefore this paper develops a quick-responsive ESC system based on the queuing theory, where each depository in the ESC is considered as a server, and waiting commodities are in a queue to accept the server's services in a three-echelon network. ESC system will be optimized to provide the relief operations under the consideration of both economic loss and response time limitation.

In this paper we develop a new quick-responsive ESC system with a queuing model in a specified three-layer network. Each depository in the ESC is modeled as a server and waiting commodities are in a queue. By using the queuing model, we can obtain the classical performances of ESC, such as average queue length and average waiting/residence time, and optimize the relief operations under the consideration of both economic loss and response time limitation.

The main contributions of this study are as follows. Firstly, a new emergency service supply chain model fulfilling the requirement of quick responses and deliveries for disaster relief is developed. Secondly, a queuing model is embedded in this model for the first time to calculate the performance of ESC. Thirdly, the physical network of ESC with a queuing model is proposed as an effective refined method to solve the integrated problem including vehicle routing problem and location problem of emergency relief centers with multiple layers.

The rest of this paper is organized as follows.  Section 2 presents the literature review on emergency management. A system specification is provided with a physical network of ESC including a queuing model in Section 3. A queuing minimal response time location-allocation model is formulated with a heuristic method in Section 4. A case study is introduced in Section 5 to demonstrate the performance of the proposed approach. Finally, Section 6 draws conclusions and provides directions for future research.

## 2. Literature Review

Emergency supply chain management is the key to the success of relief demand management under the condition of large-scale disasters. The difficulty of emergency supply chain management is rooted in the uncertainties of abrupt relief demand and collaboration of chaotic condition. Unlike demanders in business logistics, the relief demanders are the disaster-affected people but their locations and their demands may not be predicted precisely before disasters happen. Apparently, efficient relief supplying underlines the challenge of collaborative relief demand management in the emergency supply chain management. Despite the urgent necessity of collaborating relief demand management in the whole supply chain, there is no straightforward collaboration model available for the above issue. In contrast with the optimization-based demand and supply models, relief demand management must overcome more issues in disaster uncertainties and delivery collaboration.

In brief, the existing uncertainty based demand models appear unsuitable for chaotic relief demand management addressed in this study. Instead, most of the existing relief demand management models in the emergency supply chain appear to be limited to general cases for business operations. From the literature review, we illustrate several related subjects associated with typical models in the following for further discussion.

By comparing the business supply chain and the humanitarian relief chain, Beamon [10] revealed several specific characteristics of relief material supply chains, including zero lead times, high stakes, unreliable, incomplete, or nonexistent prior information, and different demand pattern. Oloruntoba and Gray [11] developed an agile supply chain model for humanitarian aid by applying practical elements of conventional supply chains to the ESC. Lodree Jr. and Taskin [12] introduced a stochastic inventory control model to prepare for potential hurricane activity and described a dynamic programming algorithm to solve the inventory problem. Bhakoo et al. [13] developed an understanding of the nature of collaborative arrangements for the management of inventories in Australian hospital supply chains.

Despite the recent emergence of emergency supply chain management that has increasingly drawn researchers' attention, most previous works appear to address the issues of relief supply and distribution contingent on relief demand assumptions. Yi and Kumar [14] decomposed the emergency logistics problem into two decision-making phases and proposed an ant colony optimization (ACO) model to solve a multicommodity network flow problem. Tzeng et al. [15] simplified the disaster context and proposed a fuzzy multiobjective programming method to optimize multiobjective functions to avoid the possibility of a severely unfair relief distribution. Chiu and Zheng [16] addressed the multiple emergency traffic flows outbound from the affected areas using a linear cell transmission model (CTM).

In order to deal with the optimization of emergency management, there are a large number of studies on developing operational research (OR) methods for it [17]. These methodologies include the use of linear programming techniques, fuzzy methods, stochastic programming models, probabilistic models, simulation, and decision theory and queuing theory [5]. Stochastic programming has been successfully used in the area related to disaster studies [18, 19]. As different approaches to handle emergency problems, simulation and decision theory have also been adopted as methodologies in this field. Queuing theory has been applied to explore emergency facility location problems recently. Shavandi and Mahlooji [20] combined fuzzy theory and queuing method to develop a maximal covering location-allocation model. Galvão and Morabito [21] applied the hypercube queuing model to solve probabilistic location problems in the emergency service system. Geroliminis et al. [22] presented a queuing model for locating emergency vehicles on urban networks considering both spatial and temporal demand characteristics such as the probability that a server is not available when required. But numerous emergency practices reveal that chaotic status after disasters worsens rescue coordination, and the relief supply chain is often blocked or congested in practices. Multiple emergency sources improve robustness of supply chains, but coordination among multiple servers is more critical for emergency management. Meanwhile organizational skills require that the emergency supply chain operated by local government consists of multiple echelons, which forms vertical coordination in emergency context. All those problems need to be resolved in new models and algorithms. However no paper indicated above embeds the queuing models into the multiechelon emergency service supply chain system.

Therefore, to resolve the issues mentioned above, we present a relief demand management model of emergency supply chain to address the above issue under the disorder and uncertain conditions in affected areas during the crucial rescue period of a large-scale disaster. Rooted in the techniques of collaboration in emergency supply chain coupled with queuing theory and system optimization, the proposed methodology embeds three mechanisms: (1) multiechelon supply chain model for disasters, (2) dynamic facility location and vehicle routing selection, and (3) rescue system management collaboration.

Relative to the previous literature, the proposed relief demand management methodology has the following two distinctive features. (1) The model is capable of collaborating urgent relief demand management in the large-scale disaster contexts and accelerating rescue efforts to save casualty loss. (2) To facilitate dynamic relief allocation and distribution, the proposed model practically groups humanitarian reliefs into multiechelon resource suppliers and distribution centers, which form an emergency response system for uncertain large disasters.

## 3. System Specification

**Figure 1:**
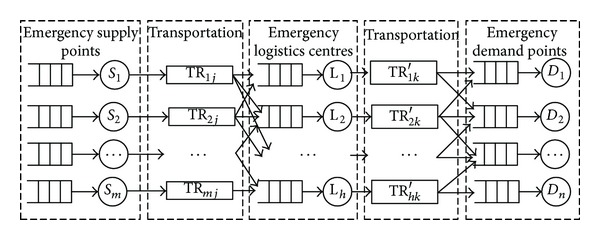
The queuing network of ESC.

An ESC system involves selection of sites and vehicle routing decisions which are two major problems in a disaster response environment. The optimal facilities locations and path selections can guarantee that the commodities will be sent from the supply depots to the demand points in affected areas as quickly as possible to maximize the survival rate of wounded persons. The above problems arouse our interests to propose queuing modeling for the ESC system. Hence, a queuing network of emergency supply chain is formulated in this paper as shown in Figure 1.

The queuing network of ESC involves a queuing flow formulation, where the three supply chain members, namely, supply points (SP), emergency logistics centers (ELCs), and demand points (DP), are treated as servers. Emergency commodities, such as food, shelter, personnel, machinery and medicine, are modeled as customers. The upstream and downstream nodes of ESC system constitute some basic activities that are producing, sorting, processing, packing, delivering, and so forth. These activities are regarded as the service for customers. Consider the following.

1. Locations of emergency supply points are in *S*_1_, *S*_2_, *S*_3_,…, *S*_*m*_.
2. Locations of emergency logistics centers are in *L*_1_, *L*_2_, *L*_3_,…, *L*_*r*_.
3. Locations of emergency demand points are in *D*_1_, *D*_2_, *D*_3_,…, *D*_*n*_.
  - Vehicle routing choices for the commodity flows in the system are considered in the following:
4. TR_1*j*_: from *S*_1_ to one of the nodes (*L*_1_, *L*_2_, *L*_3_,…, *L*_*r*_), TR_2*j*_: from *S*_2_ to one of the nodes (*L*_1_, *L*_2_, *L*_3_,…, *L*_*r*_),…, and TR_*mj*_: from *S*_*m*_ to one of the nodes (*L*_1_, *L*_2_, *L*_3_,…, *L*_*r*_), *j* = 1,2, …, *r*.
5. TR_1*k*_: from *L*_1_ to one of the nodes (*D*_1_, *D*_2_, *D*_3_,…, *D*_*n*_), TR_2*k*_: from *L*_2_ to one of the nodes (*D*_1_, *D*_2_, *D*_3_,…, *D*_*n*_), …, and TR_*rk*_: from *L*_*r*_ to one of the nodes (*D*_1_, *D*_2_, *D*_3_,…, *D*_*n*_), *k* = 1,2, …, *n*.

Each commodity is considered as a queue where batches are waiting to be serviced. The selection of sites and vehicle routing decision may be operated under the consideration of the estimated throughput response time from supply depots to demand depots in affected areas. The response time comprises not only the transportation times between upstream and downstream nodes but also the total waiting times and service times in the queuing network.

Therefore, the above three nodes in the system are assumed to behave as an *M*/*M*/1 queuing, where relief supplies are treated as customers. On the basis of the specified ESC queuing network, we adopt the following hypothesis for system operations.

1. The corresponding geographic relationships between upstream and downstream nodes are available from the existing governmental databases and the relief demand needed in a given affected area can be readily accessible via advanced disaster detection technologies.
2. The locations of emergency logistics centers are only fixed in the given alternative sites.
3. The first customer in the queue receives services firstly, namely “first come first served.”

## 4. Mathematical Formulation

Based on the aforementioned system specification, we propose a queuing theory in this section for facility location and path selection problems in a multistage emergency supply chain network. Firstly, the notations, parameters, and decision variables for the mathematical formulation are introduced. After that, the objective function for the model is established. And then the formulation of the constraints of the problems is presented.

### 4.1. The Parameters and Decision Variables

The sets, parameters, and decision variables are defined as follows.

(*1) Notations*

  
*I*: set of supply depots, *i* ∈ *I*, *i* = 1,2, 3,…*m*,
  
*J*: set of alternative sites of emergency logistics centers, *j* ∈ *J*, *j* = 1,2, 3,…, *h*,
  
*K*: set of depots for handing out relief goods, *k* ∈ *K*, *k* = 1,2, 3,…, *n*,
  
*R*: set of commodities, *r* ∈ *R*, *r* = 1,2, 3,…, *R*.

(*2) Parameters*

  
*λ*: the interarrival time of the emergency demand following a negative exponential distribution,
  
*μ*: the service rate at each node in the queuing network system,
  
*δ*: the parameter of the negative exponential distribution,
  
*c*: the lower bound of a uniformly distributed random variable that indicates the quantity of resources in a relief request,
  
*d*: the upper bound of a uniformly distributed random variable that indicates the quantity of resources in a relief request,
  
*t*_*ijk*_^*r*^: the response time of the ESC system for commodity type *r* from node *i* to node *k* going through logistics centers located at nodes *j*,
  WT^*r*^: the sojourn time of commodity type *r* in the system,
  WT_*q*_^*r*^: the waiting time of commodity type *r* in the queue,
  TR_*ij*_^*r*^: the transportation time for commodity type *r* from node *i* to node *j*,
  TR_*jk*_^*r*^: the transportation time for commodity type *r* from node *j* to node *k*,
  DTR_*ij*_: the distance between node *i* and node *j*,
  DT_*kj*_: the distance between node *j* and node *k*,
  DC: the penalty cost for unavailability of commodities demand within the maximum promised response time,
  
*a*_*j*_: the fixed costs of locating the emergency logistics center *j*,
  
*q*_1_: the total number of logistics center to be fixed,
  
*q*_2_: the number of supply depots delivering commodities to the same emergency logistic centers,
  
*q*_3_: the number of demand nodes accepting items from the same emergency logistics center,
  
*v*_*ij*_^*r*^: transport speed for commodity type *r* from node *i* to node *j*,
  
*v*_*jk*_^*r*^: transport speed for commodity type *r* from node *j* to node *k*.

(*3) Decision Variables*

(1) $$\begin{array}{c} x_{j}=\{\begin{array}{cc} 1, & \text{emergency logistics center built }\\ & \text{on the site}\;\;j,\;\\ 0, & \text{otherwise} \end{array}\\ y_{ij}=\{\begin{array}{cc} 1, & \text{relief resources from emergency supply}\\ & \text{point}\;\;i\;\;\text{transported}\;\;\text{to}\;\;\text{emergency}\\ & \text{logistics}\;\text{center}\;\;j,\;\\ 0, & \text{otherwise} \end{array}\\ z_{jk}=\{\begin{array}{cc} 1, & \text{relief}\;\;\text{resources}\;\;\text{from}\;\;\text{emergency}\;\;\text{logistics}\\ & \text{center}\;\;j\;\;\text{transported}\;\;\text{to}\;\;\text{relief}\\ & \text{demand}\;\;\text{point}\;\;k,\;\\ 0, & \text{otherwise}. \end{array} \end{array}$$

### 4.2. Queuing Minimal Unsatisfied Demand Location-Allocation Model

Three types of members (SP, ELC, and DP) involved in this system are in serial connection. The transportation routes are necessary to deliver items from upstream nodes to downstream nodes. Based on this assumption, a queuing minimal response location-allocation model for the three-stage queuing network is formulated as follows.

Objective function

(2) $$\begin{array}{c} \min Z=\underset{r\in R}{\sum }\;\underset{i\in I}{\sum }\;\underset{j\in J}{\sum }\;\underset{k\in K}{\sum }t_{ijk}^{r} \end{array}$$

subject to

(3) $$\begin{array}{c} y_{ij}^{r}\leq x_{j}^{}\;\forall i\in I,\;\;j\in J,\;\;r\in R \end{array}$$

(4) $$\begin{array}{c} z_{jk}^{r}\leq x_{j}^{}\;\forall k\in K,\;\;j\in J,\;\;r\in R \end{array}$$

(5) $$\begin{array}{c} \underset{j\in J}{\sum }y_{ij}^{r}=1\;\forall i\in I,\;\;r\in R \end{array}$$

(6) $$\begin{array}{c} \underset{j\in J}{\sum }z_{jk}^{r}=1\;\forall k\in K,\;\;r\in R \end{array}$$

(7) $$\begin{array}{c} \underset{l\in L\;|\;d_{il}\leq d_{ij}}{\sum }y_{il}^{r}\geq x_{j}^{}\;\forall i\in I,\;\;j\in J,\;\;r\in R \end{array}$$

(8) $$\begin{array}{c} \underset{l\in L\;|\;d_{lk}\leq d_{jk}}{\sum }z_{lk}^{r}\geq x_{j}^{}\;\forall k\in K,\;\;j\in J,\;\;r\in R \end{array}$$

(9) $$\begin{array}{c} \underset{}{\sum }a_{j}^{}x_{j}^{}\leq B\;\forall j\in J \end{array}$$

(10) $$\begin{array}{c} \underset{}{\sum }x_{j}^{}=q_{1}\;\forall j\in J \end{array}$$

(11) $$\begin{array}{c} \underset{i\in I}{\sum }y_{ij}^{r}=q_{2}\;\forall j\in J,\;\;r\in R \end{array}$$

(12) $$\begin{array}{c} \underset{k\in K}{\sum }z_{jk}^{r}=q_{3}\;\forall j\in J,\;\;r\in R \end{array}$$

(13) $$\begin{array}{c} t_{ijk}^{r}=\text{W}{\text{T}}_{i}^{r}+\text{W}{\text{T}}_{j}^{r}+\text{W}{\text{T}}_{k}^{r}+\text{T}{\text{R}}_{ij}^{r}+\text{T}{\text{R}}_{jk}^{r} \end{array}$$

(14) $$\begin{array}{c} x_{j}^{r},y_{jk}^{r},z_{ij}^{r}=0,1\;\forall k\in K,\;\;i\in I,\;\;j\in J,\;\;r\in R. \end{array}$$

The objective aims at minimizing the mean system response time of relief resources including the sojourn time in the queuing network system and transportation time. Constraints (3) and (4) ensure that deliveries can only be made if emergency logistics centers are fixed. Constraint (5) enforces that the items from the supply nodes can only be delivered to one logistics center and constraint (6) enforces that every demand nodes can obtain items from just one logistics center. Constraints (7) and (8) represent that the items from upstream nodes are transported to the nearest downstream nodes in the queuing network system. Constraint (9) limits the sum of fixed costs of locating the emergency logistics centers. Constraint (10) shows that the total number of logistics center to be sited is equal to *q*_1_. Constraint (11) forces that the number of supply depots delivering commodities to the same emergency logistic centers is equal to *q*_2_ to ensure that each emergency logistics is with enough capacity to deal with these commodities. Constraint (12) forces that the number of demand nodes accepting items from the same emergency logistics center is equal to *q*_3_ to ensure that the items from each emergency logistics center are enough to satisfy the emergency request. Constraint (13) represents that the response time is equal to the sojourn time plus the transporting time. Constraint (14) defines all the decision variables to be binary integer variables.

Next, we use the queuing theory to compute the objective function. The emergency supply chain system is a series-parallel hybrid queuing system consisting of three service nodes. We can assume that the relief requests for the emergency demand depots follow a Poisson distribution with intensity *λ*_*k*_. Each emergency logistics center serves a set of demand points, and therefore the relief requests for an emergency service at the logistics center are the union of the relief requests of the nodes in the set. Therefore, they can be depicted as a stochastic process equal to the sum of several Poisson processes, with an intensity *λ*_*j*_′ equal to the sum of the intensities of the processes at the nodes served by the logistics center. We can rewrite parameter *λ*_*j*_′ by using variables *z*_*jk*_:

(15) $$\begin{array}{c} \lambda_{j}^{\prime }=\overset{n}{\underset{k=1}{\sum }}\lambda_{k}z_{jk}. \end{array}$$

The relief request for the supply depots is also assumed to follow a Poisson distribution with intensity *λ*_*i*_′′ and also similar equilibrium equations exist between the arrival rate of the relief request for the supply depots and for the emergency logistics centers. For the sake of simplicity, we assume that the arrival rate of the relief request for the supply depots that transport emergency resources to the same emergency logistics center has the same value. Thus the parameter *λ*_*i*_′′ can be rewritten by using variables *y*_*ij*_ and the constant *q*_2_:

(16) $$\begin{array}{c} \lambda_{i}^{\prime \prime }=\frac{1}{q_{2}}\overset{h}{\underset{j=1}{\sum }}\lambda_{j}^{\prime }y_{ij}=\frac{1}{q_{2}}\overset{h}{\underset{j=1}{\sum }}\;\overset{n}{\underset{k=1}{\sum }}\lambda_{k}y_{ij}z_{jk}. \end{array}$$

**Figure 2:**
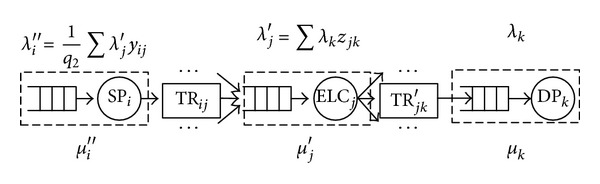
Equivalent queue of studied ESC network.

Based on the above analysis, the equivalent queue of the studied ESC network is shown in Figure 2.

From the affected people's point of view, the ESC system is equivalent to a queue network that is receiving emergency relief orders. These relief request orders are waiting to be served. The service is the process of production, collection, and processing, and the results are emergency relief resources, items, and so forth.

Emergency relief orders are characterized by (i) occurrence, (ii) quantity, and (iii) delay. Consider the following:

  
*U*: random variable indicating the occurrence time of a relief request,
  
*V*: random variable indicating the quantity of resources in every relief request,
  
*W*: UV indicating the occurrence time along with the quantity of resources in every relief request.

Assume that *U* follows a negative exponential distribution with intensify *f*_*U*_(*u*) and *V* is a uniformly distributed random variable with intensify *f*_*V*_(*v*) between *c* and *d*  (*d* > *c*). And *f*_*U*_(*u*) and *f*_*V*_(*v*) are independent. Thus,

(17) $$\begin{array}{c} f_{U}\left(u\right)=\{\begin{array}{cc} \delta e^{-\delta u}; & u\geq 0,\\ 0; & u<0, \end{array}\\ f_{V}\left(v\right)=\{\begin{array}{cc} \frac{1}{d-c}; & c<v<d,\\ 0; & \text{otherwise}, \end{array}\\ E\left(W\right)=E\left(UV\right)=E\left(U\right)E\left(V\right)=\frac{c+d}{2\delta }. \end{array}$$

Therefore, the interarrival times of the emergency demand (occurrence and quantity) follow a negative exponential distribution with intensity *λ* equal to 1/*E*(*W*). The service rate at each node in the queuing network system is an independent identically distributed random variable with intensity *μ* and the service time is 1/*μ*.

So, the interarrival time *λ* and the traffic intensity of the system *ρ* are represented as

(18) $$\begin{array}{c} \lambda =\frac{1}{E\left(W\right)}=\frac{2\delta }{c+d}, \end{array}$$

(19) $$\begin{array}{c} \rho =\frac{\lambda }{\mu }=\frac{1}{\mu E\left(W\right)}=\frac{2\delta }{\mu \left(c+d\right)}.\;\;\; \end{array}$$

Let us assume that there exits just one server at each service node and the servers are independent, which means that the queuing model at each server is an *M*/*M*/1. Then the probability distribution function of the sojourn time WT (defined as the waiting time plus the service time for a customer) in an *M*/*M*/1 queue can be presented as

(20) $$\begin{array}{c} f_{\text{WT}}\left(t\right)=\left(\mu -\lambda \right)e^{-(\mu -\lambda )t}. \end{array}$$

From (7), the cumulative distribution function of WT is

(21) fWT(t)=P(WT≤t)=∫0t(μ−λ)e−(μ−λ)tdt=1−e−(μ−λ)t.

The average sojourn time WT is given by

(22) WT=E(WT)=∫0∞fWT(t)t dt=∫0∞(μ−λ)e−(μ−λ)tt dt=1μ−λ.

Let WT_*q*_ denote the waiting time in the queue. The average waiting time is computed as

(23) $$\begin{array}{c} \text{W}{\text{T}}_{q}=\text{WT}-\frac{1}{\mu }=\frac{\lambda }{\mu \left(\mu -\lambda \right)}.\;\;\; \end{array}$$

From the well-known Little's theorem, the average customers LR in the system including the number of customers both waiting in the queue and served in the server is given by

(24) $$\begin{array}{c} \text{LR}=\lambda \text{WT}=\frac{\lambda }{\mu -\lambda }.\;\;\;\; \end{array}$$

And LR_*q*_ denote the queuing length in the system which is presented as

(25) $$\begin{array}{c} \text{L}{\text{R}}_{q}=\lambda \text{W}{\text{T}}_{q}=\frac{\lambda^{2}}{\mu \left(\mu -\lambda \right)}.\;\; \end{array}$$

From (17), (16), (18), (22), (23), (24), and (25), the average sojourn time, waiting time in the queue, the average customers including the number of customers both waiting in the queue and served in the server, and the queuing length for the ESC network system are given as

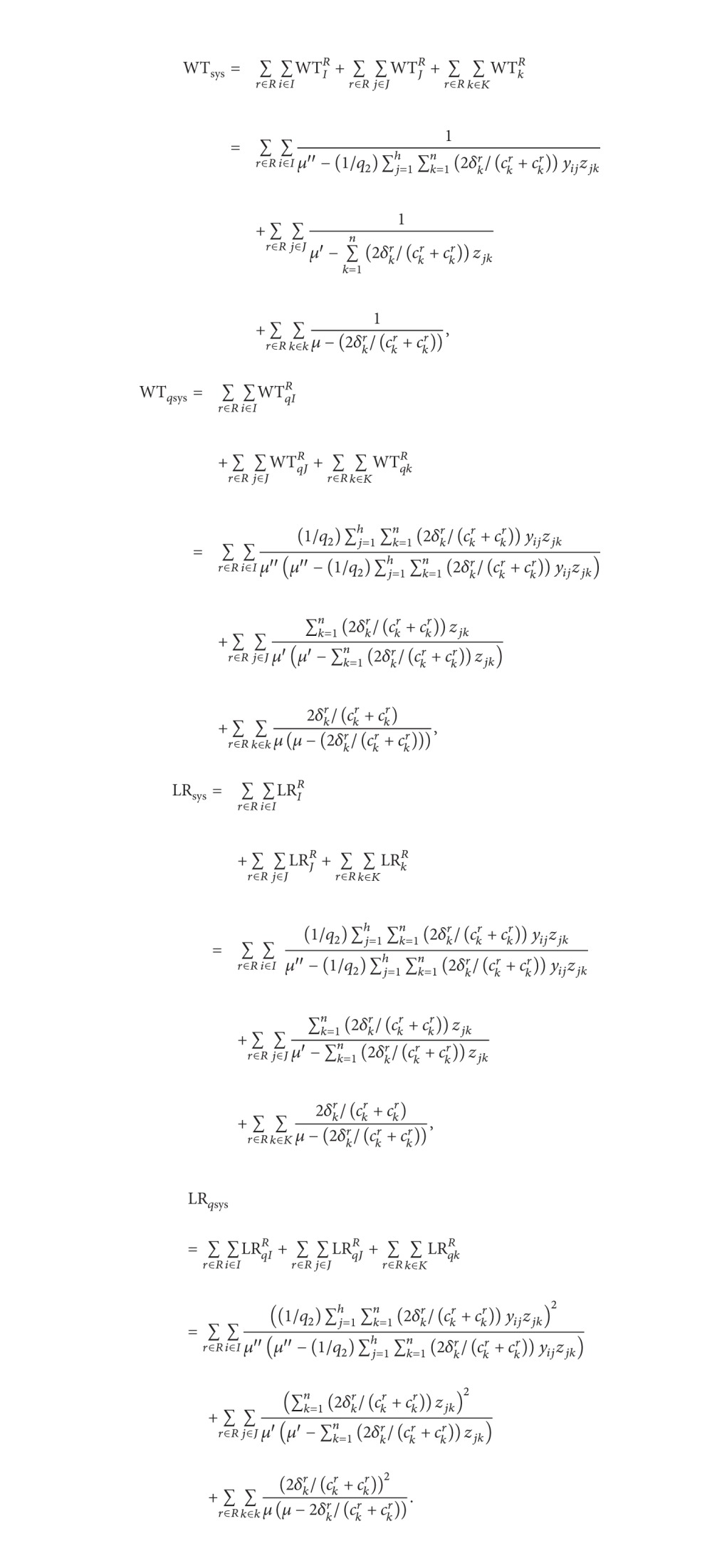
(26)


As the response time includes not only the sojourn time of emergency demand in the queuing network system but also the transportation time, therefore, when the queuing theory is applied to the present model, the above objective function can be presented as follows:

(27) min⁡⁡Z =min⁡∑r∈R ∑i∈I ∑j∈J ∑k∈Ktijkr =min⁡(∑r∈R ∑i∈I(1)     ×(μ′′−(1q2)∑j=1h∑k=1n(2δkr/(ckr+ckr))yijzjk)−1    +∑r∈R ∑j∈J1μ′−∑k=1n(2δkr/(ckr+ckr))zjk    +∑r∈R ∑k∈k1μ−(2δkr/(ckr+ckr))    +∑r∈R ∑i∈I ∑j∈JTRijryij+∑r∈R ∑j∈J ∑k∈KTRjkrzjk).

### 4.3. GA-Based Approach

**Figure 3:**
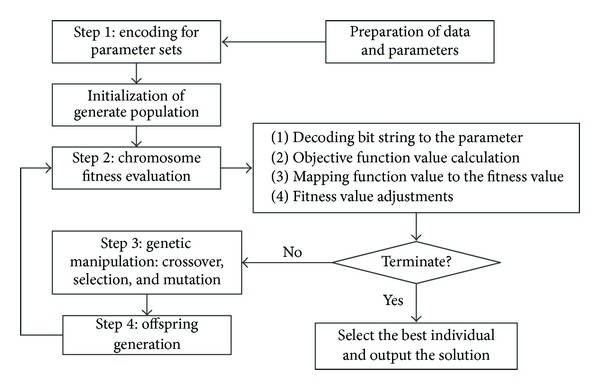
Steps of the proposed GA heuristic.

As the facility location-allocation problem is NP- (nondeterministic polynomial-) hard where the proposed 0-1 nonlinear integer programming model in this study is used, some heuristic algorithms are required to solve the problem quickly. The genetic algorithms (GAs) have been applied to location problems since 1986 by Hosage and Goodchild [23]. GA has exhibited inherent advantages such as their robust performance when solving combinatorial problems, as well as their ability to incorporate procedures and logical conditions and to handle discrete variables and nonlinear constraints in a straightforward manner [24]. These qualities make GAs particularly attractive for potential combination with other methods and external procedures. We develop a refined genetic algorithm to solve the aforementioned problem as indicated below. The associated framework for embedding the GA with emergency queue network is presented in Figure 3.

#### 4.3.1. Methodology of Genetic Algorithm

In a genetic algorithm, a population of candidate solutions to an optimization problem is evolved toward better solutions. Each candidate solution has a set of properties (its chromosomes or genotype) which can be mutated and altered; solutions are represented in binary as strings of 0 s and 1 s, but other encodings are also possible. The evolution usually starts from a population of randomly generated individuals and happens in generations. In each generation, the fitness of every individual in the population is evaluated; the more fit individuals are stochastically selected from the current population, and each individual's genome is modified (recombined and possibly randomly mutated) to form a new population. The new population is then used in the next iteration of the algorithm. Commonly, the algorithm terminates when either a maximum number of generations have been produced or a satisfactory fitness level has been reached for the population. Once the genetic representation and the fitness function are defined, a GA proceeds to initialize a population of solutions and then to improve it through repetitive application of the mutation, crossover, inversion, and selection operators.

#### 4.3.2. Procedure of the Refined Genetic Algorithm

**Step 1 (encoding)**

We suppose that the genotype of a chromosome with some genes indicates an individual corresponding to the location pattern of emergency logistics centers and paths between nodes in the system network. The gene presented by 1 indicates that an emergency logistics center is built at this location or the path between nodes in the system network is selected. The gene presented by 0 indicates that no emergency logistics centers is built there or the path between nodes in the system network which is selected is not selected. *N* chromosomes are randomly generated, and the initial population (Pop) is set.

**Step 2 (fitness evaluation)**

Once the initial population is available, evaluation of each chromosome takes place to explore the quality of the corresponding solution. For individuals with larger fitness value to be transmitted to the next generation with higher probability, the chromosome fitness function in this paper can be described as follows and *c*_max⁡_ is the maximum estimation value of min⁡⁡*Z*:

(28) $$\begin{array}{c} \text{Fit}\left(\min Z\right)=\{\begin{array}{cc} C_{\max}-\min Z, & \text{if}\;\min Z<C_{\max},\\ 0, & \text{otherwise}. \end{array} \end{array}$$

**Step 3 (crossover, selection, and mutation)**

*N* chromosomes are crossed according to a single point method with crossover probability (which is *P*_*c*_ = 0.9) to generate the offspring chromosomes. There are a total of 2*N* chromosomes for both parent and offspring chromosomes. Then the duplicate chromosomes are deleted to produce the new population (Pop). Select the first *N* chromosomes of the population on the basis of the fitness values in ascending order. When the number of chromosomes in the new population (Pop) is less than *N* (*N* = 200), *N*-|Pop| chromosomes are randomly generated (|Pop| is the number of chromosomes in the population (Pop)). Select a chromosome randomly and apply the mutation operator applied on it with probability *P*_*m*_ = 0.01.

**Step 4**

Choose new chromosomes by the roulette wheel method. Repeat Steps 3 and 4 until there are no solution improvements within a specified number of iterations.

## 5. A Case Study

**Figure 4:**
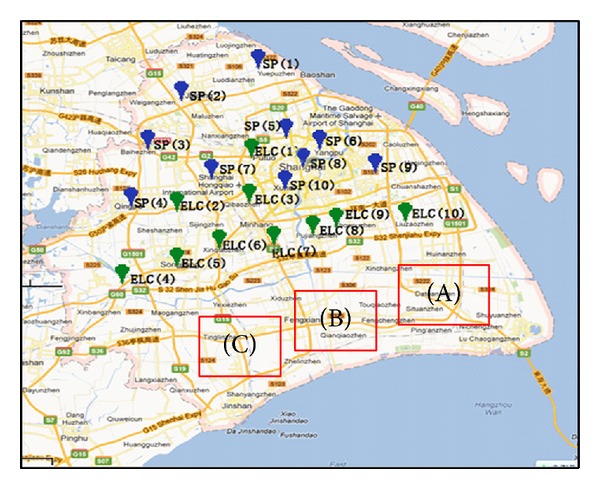
The location of facilities in an ESC system.

A case study example is presented in this section to illustrate the efficiency of our approach to realize the quick-responsive emergency relief during large-scale natural disasters. The proposed method is implemented on a scenario where a severe typhoon strikes the southeast coast of Shanghai with a great probability within the next 100 years. It is assumed that there are three regions (A, B, and C) slashed by the typhoon shown in Figure 4. One region is Lingangxincheng District (A), another one is Fengxian District (B), and the third one is Jinshan District (C).

There are ten supply points (SP): (1) disaster relief supplies reserve center in Baoshan District, (2) Automobile City in Jiading, (3) Red Cross disaster preparedness warehouse in Jiading District, (4) disaster relief supplies reserve center in Qingqu District, (5) disaster relief supplies reserve center in Putuo District, (6) disaster relief supplies reserve center in Yangpu District, (7) disaster relief supplies reserve center in Minhang District, (8) disaster relief supplies reserve center in Hongkou District, (9) disaster relief supplies reserve center in Pudong District, and (10) disaster relief supplies reserve center in Xuhui District. Based on the study of large-scale logistics center in Shanghai, we select ten potential points as the emergency logistics centers (ELCs). Four potential ELCs are prepared for the affected areas in Lingangxincheng District, two potential ELCs are planned to serve Fengxian District, and two potential ELCs are expected to serve the Jinshan District. We assume that a total of thirteen areas (DP) are seriously affected and need emergency rescue urgently, in which six affected areas are in Lingangxincheng, four affected areas are in Fengxian District, and three affected areas are in Jinshan District.

For simplicity, only one type of emergency supply resources will be considered in the queuing network. We assume that the travel speed is fixed to be equal to 40 miles per hour for all the vehicles travelling between SP and ESC. However, the travel speed between ESC and DP is assumed to be 30 miles per hour. Let us adopt the hypothesis as the above that the occurrence time of a relief request follows a negative exponential distribution with intensify *δ*, and the quantity of resources in every relief request is uniformly distributed with intensify *f*_*V*_(*v*) that the lower bound (LB) is *c* and the upper bound (UB) is *d*  (*d* > *c*). The disaster-related information including the population of the affected areas and the distance between supply point and emergency logistics center and distance between emergency logistics center and demand point are shown in Tables 1, 2, 3, and 4, respectively.

In this case study, the fixed cost to locate the emergency logistics center is assumed as *a*_1_ = 85, *a*_2_ = 100, *a*_3_ = 90, *a*_4_ = 85, *a*_5_ = 95, *a*_6_ = 110, *a*_7_ = 70, *a*_8_ = 80, *a*_9_ = 115, and *a*_10_ = 120. The total cost to build the emergency logistics centers is assumed not exceeding to be *B* = 500. For simplicity of the computation, we presume that *q*_1_ = 5, *q*_2_ = 2, and *q*_3_ = 3.

According to the background of the above problem, a simplified 10 × 10 × 15 emergency supply chain network is developed in the assumed scenario, where the geographical relationships among these supply depots, emergency logistics centers and demand points are specified. Then we apply the aforementioned model into the emergency supply chain network system. And the GA-based approach demonstrated before is coded to verify the validity of the model in Matlab2009a.

**Figure 5:**
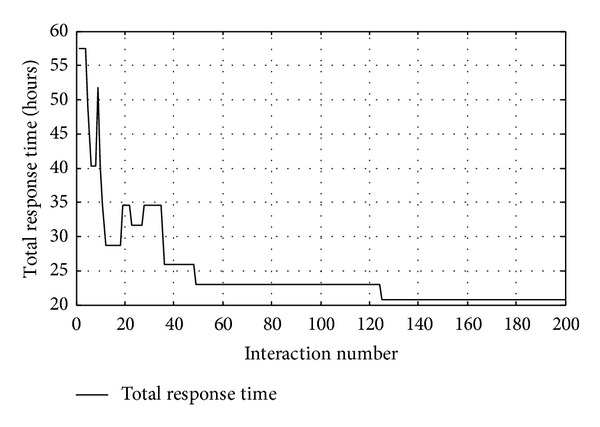
Optimization of the fitness function.

The optimization process of the fitness function is depicted in Figure 5, where the optimal response time of the queuing network is 20.71 hours.

Therefore, the average response time of each demand point is 1.38 h, which is within a reasonable range. The calculation result shows that this model can play an important role in the emergency supply chain management. Different from the general supply chain management, the timesaving is the most significant feature of the emergency supply chain system. The efficient allocation of emergency resources to demand points improves not only the operational performance of the emergency supply chain system in the emergency supply side but also the affected people's survival rate in the disaster areas. From the psychology of point of view, quick responses and deliveries for disaster relief not only may improve the governmental efforts to rescue but also strengthen the affected people's willpower to be alive, hence stabilizing the affected areas.

The emergency supply chain system is composed of three members, which have different functions in the relief supply delivery process. And the upstream nodes and downstream nodes in the ESC system are connected by roads. If we treat each node in the system as the node in a network, consider the roads as the arcs of the network, and treat the length of each road as the weight of each arc, then the ESC system can be seen as a directed network diagram with weights. And the response time of the system includes two parts: the first part is the transportation time of the relief supply request on the roads and the second part is the sojourn time of the relief supply request in each node.

**Figure 6:**
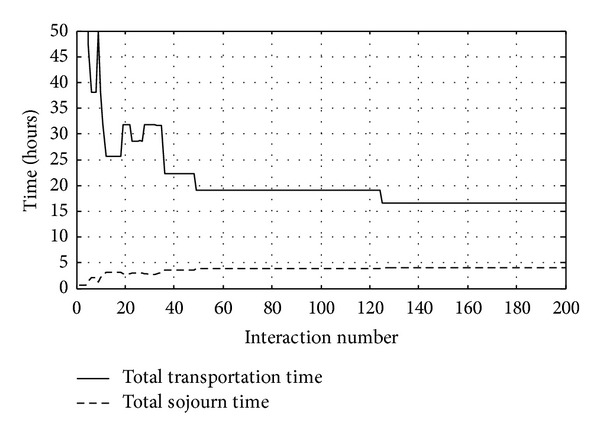
Optimization of total transportation time and total sojourn time.

Figure 6 indicates the values of the above two components of response time, with the total transportation time being 16.64 h and the total sojourn time being 4.07 h. This iterative process also demonstrates the convergence of the heuristic algorithm.

**Figure 7:**
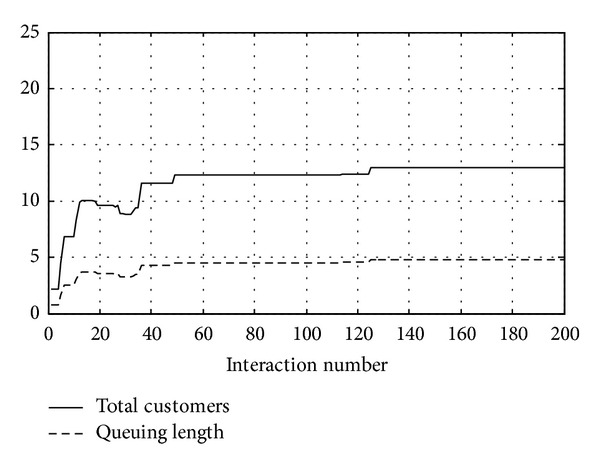
Optimization of the total customers and the queue length.

Queuing models are effective tools for obtaining information about important performance of each facility in the ESC system like sojourn times, waiting times, queue lengths, total customers in the system, and so on. In order to analyze the efficiency of facilities in the system, we calculate the total customers and the queue lengths which are shown in Figure 7. We can see that the solution of the above model will not create queuing delays of relief supply so that the losses generated by the queuing delay can be avoided. This iterative process also illustrates the robustness and the potential applicability of our model.

Based on the built network, the coordination between the upstream node and the downstream node in this system can be enforced and the performance of basic activities including producing, sorting, processing, packing, and delivering can be improved to minimize the response time of the system. The emergency logistics centers for the system should be located in DP(6), DP(7), DP(8), DP(9), and DP(10). And the optimal paths are {SP(1), SP(6) → ELC(8) → DP(1), DP(2), DP(9)}, {SP(2), SP(4) → ELC(6) → DP(13), DP(14), DP(15)}, {SP(3), SP(8) → ELC(7) → DP(10), DP(11), DP(12)}, {SP(5), SP(9) → ELC(10) → DP(3), DP(4), DP(5)}, and {SP(7), SP(9) → ELC(9) → DP(6), DP(7), DP(8)}. Overall, the examination reveals that the proposed method can be a useful tool for a quick response to the urgent need of relief in the large-scale disaster-affected areas.

## 6. Conclusions

An emergency supply chain (ESC) system is developed in this paper for the quick response to the urgent need of relief in the large-scale disaster-affected areas, which mainly consists of three chain members in series: supply points (SP), emergency logistics centers (ELCs), and demand points (DP). As the timeliness is the most crucial characteristic of the ESC system and queuing theory is a useful tool in the analysis of the performance of the time-dependent system, we propose queuing modeling for the ESC system to optimize the emergency rescue process. Based on the above queuing network system, a queuing minimal response time location-allocation model is established to decide the selection of emergency logistics centers and vehicle routing decision. For the complexity of mathematical model, the GA-based approach is introduced to solve the model.

A case study with an assumed severe typhoon striking the southeast coast of Shanghai with a great probability within the next 100 years is conducted to assess and validate our model. The optimal response time of the queuing network is 20.71 hours and the average response time of each demand point is 1.38 h, which is within a reasonable range. The optimal emergency logistics centers for the system are DP(6), DP(7), DP(8), DP(9), and DP(10). Besides, the performance of queuing modeling for the ESC system illustrates the robustness and the potential applicability of our model.

For future research, we suggest the following three directions. Firstly, we will investigate the queuing modeling under consideration of the facility blocking problems and demand priority for the emergency relief points. Secondly, we will study the generation of emergency demand and service time obeying the other random distributions. Thirdly, more efficient multiobjective optimization methodologies for both the above problem and emergency resources transportation scheduling problem will be considered.

## Figures and Tables

**Table 1:** Population and demand data of the affected areas.

Affected area	Population	(*c*, *d*)	*δ*
Area 1 (Zhuqiaozhen)	105,807	(4, 12)	16
Area 2 (Huinanzhen)	213,845	(4, 12)	16
Area 3 (Laogangzhen)	37,408	(5, 14)	18
Area 4 (Datuanzhen)	71,162	(4, 12)	16
Area 5 (Xingangzhen)	21,475	(6, 16)	20
Area 6 (Shuyuanzhen)	59,831	(6, 16)	20
Area 7 (Luchaogangzhen)	27,850	(6, 16)	20
Area 8 (Situanzhen)	65,389	(5, 14)	18
Area 9 (Qingcunzhen)	89,163	(5, 14)	18
Area 10 (Nanqiaozhen)	361,185	(3, 10)	14
Area 11 (Zhuanghangzhen)	62,388	(3, 10)	14
Area 12 (Zhelinzhen)	62,589	(3, 10)	14
Area 13 (Caojingzhen)	40,722	(2, 8)	12
Area 14 (Shanyangzhen)	84,640	(2, 8)	12
Area 15 (Jinshanzhen)	70,815	(2, 8)	12

**Table 2:** The service rates specifications of different servers in the network.

SP	*μ*′′_1_	*μ*′′_2_	*μ*′′_3_	*μ*′′_4_	*μ*′′_5_	*μ*′′_6_	*μ*′′_7_	*μ*′′_8_	*μ*′′_9_	*μ*′′_10_					
	10	9	11	8	11	12	14	16	14	17					

ELC	*μ*′_1_	*μ*′_2_	*μ*′_3_	*μ*′_4_	*μ*′_5_	*μ*′_6_	*μ*′_7_	*μ*′_8_	*μ*′_9_	*μ*′_10_					
	16	18	11	10	11	12	14	16	14	10					

DP	*μ*_1_	*μ*_2_	*μ*_3_	*μ*_4_	*μ*_5_	*μ*_6_	*μ*_7_	*μ*_8_	*μ*_9_	*μ*_10_	*μ*_11_	*μ*_12_	*μ*_13_	*μ*_14_	*μ*_15_
	8	8	8	8	10	9	11	8	9	12	9	8	12	14	13

**Table 3:** The distance between supply point and emergency logistics center.

	SP(1)	SP(2)	SP(3)	SP(4)	SP(5)	SP(6)	SP(7)	SP(8)	SP(9)	SP(10)
ELC(1)	18.28	17.07	11.97	21.63	6.14	8.26	10.91	9.73	15.30	8.79
ELC(2)	30.73	21.47	16.24	5.71	20.39	20.56	8.02	16.79	25.54	13.90
ELC(3)	22.71	22.88	17.12	16.22	14.19	14.63	9.98	11.02	18.42	7.56
ELC(4)	44.22	30.95	24.62	12.64	39.71	34.92	24.29	32.76	41.53	30.03
ELC(5)	39.62	28.01	27.16	15.07	32.56	27.69	14.49	25.51	33.25	20.06
ELC(6)	35.85	23.56	19.23	16.47	27.84	23.13	9.89	21.36	28.39	14.37
ELC(7)	29.73	29.68	23.64	23.01	20.36	21.49	12.75	17.97	25.75	14.71
ELC(8)	23.08	32.61	28.78	28.92	14.94	14.01	17.64	11.32	19.61	19.33
ELC(9)	25.84	36.43	33.03	32.49	20.15	11.65	22.58	9.41	16.00	24.19
ELC(10)	30.38	46.66	42.40	41.39	28.31	20.39	30.08	17.42	14.05	31.05

**Table 4:** The distance between emergency logistics center and demand point.

	ELC(1)	ELC(2)	ELC(3)	ELC(4)	ELC(5)	ELC(6)	ELC(7)	ELC(8)	ELC(9)	ELC(10)
DP(1)	37.61	41.91	32.53	45.99	38.01	37.52	24.39	23.97	15.53	7.39
DP(2)	42.71	47.02	37.61	47.94	39.45	41.52	28.18	28.22	18.98	10.75
DP(3)	45.08	48.86	39.86	50.28	45.01	43.84	30.46	30.25	21.17	13.56
DP(4)	43.64	45.79	36.97	48.24	43.07	41.38	27.98	29.40	19.56	17.10
DP(5)	52.83	54.16	43.86	54.66	54.35	49.86	38.36	32.71	26.93	19.17
DP(6)	52.14	57.21	48.66	58.96	52.94	52.07	37.97	42.21	32.52	25.53
DP(7)	53.34	57.76	52.99	58.62	53.59	51.64	38.71	38.51	29.55	25.54
DP(8)	46.89	45.81	37.63	49.83	43.06	41.73	28.18	29.97	22.00	19.68
DP(9)	33.09	36.24	26.57	38.34	33.34	31.54	18.86	31.54	34.52	32.35
DP(10)	26.78	32.63	19.99	29.11	27.41	25.18	11.98	25.05	23.60	30.37
DP(11)	33.69	29.12	23.86	23.49	21.82	18.58	18.94	23.92	29.15	35.96
DP(12)	33.76	37.94	25.41	32.87	32.75	26.12	17.08	30.62	28.96	32.56
DP(13)	36.91	34.19	29.87	28.96	26.94	22.79	21.62	35.00	32.75	40.07
DP(14)	42.30	32.73	34.02	30.11	25.55	21.41	26.06	39.06	37.49	44.48
DP(15)	46.09	37.65	38.58	25.33	30.54	26.80	31.66	43.90	42.49	48.97
